# Evaluation of Cellular Phenotypes Implicated in Immunopathogenesis and Monitoring Immune Reconstitution Inflammatory Syndrome in HIV/Leprosy Cases

**DOI:** 10.1371/journal.pone.0028735

**Published:** 2011-12-21

**Authors:** Carmem Beatriz Wagner Giacoia-Gripp, Anna Maria Sales, José Augusto da Costa Nery, Joanna Reis Santos-Oliveira, Ariane Leite de Oliveira, Euzenir Nunes Sarno, Mariza Gonçalves Morgado

**Affiliations:** 1 Laboratory of AIDS and Molecular Immunology, Oswaldo Cruz Institute, Oswaldo Cruz Foundation, Rio de Janeiro, Brazil; 2 Leprosy Laboratory, Oswaldo Cruz Institute, Oswaldo Cruz Foundation, Rio de Janeiro, Brazil; 3 Laboratory of Interdisciplinary Medical Research, Oswaldo Cruz Institute, Oswaldo Cruz Foundation, Rio de Janeiro, Brazil; Tulane University, United States of America

## Abstract

**Background:**

It is now evident that HAART-associated immunological improvement often leads to a variety of new clinical manifestations, collectively termed *immune reconstitution inflammatory syndrome*, or IRIS. This phenomenon has already been described in cases of HIV coinfection with *Mycobacterium leprae*, most of them belonging to the tuberculoid spectrum of leprosy disease, as observed in leprosy reversal reaction (RR). However, the events related to the pathogenesis of this association need to be clarified. This study investigated the immunological profile of HIV/leprosy patients, with special attention to the cellular activation status, to better understand the mechanisms related to IRIS/RR immunopathogenesis, identifying any potential biomarkers for IRIS/RR intercurrence.

**Methods/Principal Findings:**

Eighty-five individuals were assessed in this study: HIV/leprosy and HIV-monoinfected patients, grouped according to HIV-viral load levels, leprosy patients without HIV coinfection, and healthy controls. Phenotypes were evaluated by flow cytometry for T cell subsets and immune differentiation/activation markers. As expected, absolute counts of the CD4+ and CD8+ T cells from the HIV-infected individuals changed in relation to those of the leprosy patients and controls. However, there were no significant differences among the groups, whether in the expression of cellular differentiation phenotypes or cellular activation, as reflected by the expression of CD38 and HLA-DR. Six HIV/leprosy patients identified as IRIS/RR were analyzed during IRIS/RR episodes and after prednisone treatment. These patients presented high cellular activation levels regarding the expression of CD38 in CD8+ cells T during IRIS/RR (median: 77,15%), dropping significantly (*p*<0,05) during post-IRIS/RR moments (median: 29,7%). Furthermore, an increase of cellular activation seems to occur prior to IRIS/RR.

**Conclusion/Significance:**

These data suggest CD38 expression in CD8+ T cells interesting tool identifying HIV/leprosy individuals at risk for IRIS/RR. So, a comparative investigation to leprosy patients at RR should be conducted.

## Introduction

The introduction of highly active antiretroviral therapy (HAART) for HIV infection in the mid-1990s is associated with clinical benefits, including the resolution of opportunistic infections and malignancies and the decline in hospitalizations and mortality rates [1]. These benefits are, in part, associated with the suppression of HIV viremia and the improvement of the immune function, as indicated by increases in total CD4+ T cell counts [2]. However, it is now also evident that the HAART-associated increase in immunity often leads to a variety of new clinical manifestations as a result of the dysregulation of the immune system or an inflammatory response to both intact subclinical pathogens and residual antigens [3]–[5]. These manifestations have been collectively termed immune reconstitution inflammatory syndrome (IRIS) because the phenomenon occurs during HAART-induced immune recovery and involves a host inflammatory response [6]. An increasing number of conditions are reported as IRIS events, and these most frequently occur in conjunction with mycobacterial (tuberculosis or *Mycobacterium avium complex* infection) and cryptococcal diseases [7]. Prior to the HAART era, there was ample appreciation that improvements in the immune function could result in pathological inflammation. These so-called paradoxical reactions were well described among non-HIV-infected patients treated for *Mycobacterium tuberculosis* infection [8]. Clinical worsening in these patients following the initiation of anti-*M. tuberculosis* therapy had been attributed to a reversal of the immunosuppression induced by this infection.

Despite numerous descriptions of IRIS conditions, its pathogenesis remains largely unknown. It is now accepted, however, that it involves a combination of immune restoration activities following HAART, an underlying antigenic burden, and host genetic susceptibility [7].

The presence of an antigenic stimulus for the development of the syndrome appears to be necessary and may derive from an infectious or noninfectious agent. The source of this antigenic stimulus could be intact, “clinically silent” organisms or dead or dying organisms and their residual antigens [4], [5]. Although uncommon, our group and others have previously described the IRIS phenomenon in cases of HIV coinfection with *Mycobacterium leprae* [9]–[14]. The majority of these reported cases belong to the tuberculoid spectrum of leprosy, implying that a good host immune response is involved in this pathological condition. The coexisting type I leprosy reaction in these individuals further supports the idea that the recovery of the immunological system causes this paradoxical response. Similarly to *M. tuberculosis*, leprosy reaction most often occurs during multidrug chemotherapy in both paucibacillary and multibacillary patients [15]. Paucibacillary cases develop a type 1 or leprosy reversal reaction (RR) while a type 2 reaction develops in multibacillary patients. In RR, the level of cell-mediated immunity against *M. leprae* is suddenly elevated, resulting in an inflammatory response in the skin and nerves affected by the disease. Inflammatory reactions during treatment are, therefore, routine in *M. Leprae*-infected patients.

The pathogenic mechanism receiving the most attention involves the theory that IRIS is precipitated by the extent of immune restoration following HAART. Some studies suggest that differences in baseline CD4+ cell numbers at the beginning of HAART are responsible [16], [17]. Alternatively, immunological mechanisms may involve qualitative changes in lymphocyte function or phenotypic expression, e.g., an increase in memory CD4+ cells, which are primed to recognize previous antigenic stimuli [18]. Therefore it can be deduced that other immunological factors may be involved in the pathogenesis of IRIS. Chronic immune activation is a characteristic of HIV disease progression, which is strongly correlated to HIV RNA levels that decrease as a result of HAART-induced virological suppression [19]–[21]. In fact, immune activation has been considered an important cause of HIV pathogenesis [22], [23]. Differential expression of cellular activation markers during IRIS/leprosy reversal reaction (IRIS/RR) episodes should reflect the risk of developing this paradoxical disease.

This study was designed to evaluate the immunological profile of HIV/leprosy patients, paying particular attention to cellular activation states, to better understand the mechanisms leading to IRIS/RR immunopathogenesis. We also aimed to identify any potential parameters such as immunophenotypic markers associated with the occurrence of IRIS/RR to enable the development of diagnostic criteria and prevention strategies. A clear understanding of IRIS pathogenesis is required to investigate its cause, recognize which individuals are at risk, and develop effective treatment strategies.

## Methods

### Ethics Statement

Protocol was approved by the Oswaldo Cruz Foundation Ethics Committee, which is affiliated to the Brazilian National Ethics Council, under protocol no. 440/08. Written informed consent was provided by each individual participant before sample collection and study procedures began.

### Study Groups

Eighty-five individuals were assessed in this study, 42 of whom were HIV/leprosy coinfected. Seven leprosy patients (without HIV coinfection), 20 healthy individuals, and 16 previously-studied HIV patients [24] (without leprosy coinfection) were included as controls. In accordance with the Brazilian Ministry of Healthy Consensus Therapy guidelines, all HIV-infected patients were under a 3-or-more antirretrovial drug regimen at the time of enrollment, either with or without protease inhibitors, except for 7 HIV/leprosy coinfected individuals,. The leprosy and HIV/leprosy patients were followed at the Souza Araújo Out-patient Unit (Oswaldo Cruz Institute, Fiocruz, Rio de Janeiro, RJ, Brazil).

Because our focus was the immunological profile of the patients, especially their immune activation status [20], [21], which may alter as a result of HIV replication and viral load levels, the HIV/leprosy-coinfected individuals were first grouped according to their HIV viral load levels regardless of HAART status. The patients were classified as belonging to group 1 when their viral load (VL) levels were <80 copies/ml (n = 24), as group 2 when their VL levels were ≥80<10,000 copies/ml (n = 12), and group 3, when the patients presented VL levels ≥10,000 copies/ml (n = 6). Similarly, HIV-monoinfected individuals were divided into 2 groups: the undetectable VL group whose VL levels were the limit of detection (VL<LD), and the detectable VL group (VL≥LD) whose VL values were ≥80 copies/ml.

In light of their clinical leprosy status, HIV/leprosy patients were classified by way of Ridley and Jopling criteria in the absence of any RR symptoms as IRIS/RR at the time of RR, and post-IRIS/RR at the completion of treatment with glycocorticoid prednisone 6 months afterward. The 7 leprosy patients were also evaluated according to Ridley-Jopling criteria.

### Viral Load Evaluation

Quantification of the plasma viral load was determined for all HIV/leprosy coinfected patients at each visit using nucleic acid sequence-based amplification (NASBA, Organon Teknika, Boxtel, The Netherlands), which has a lower detection limit of 80 copies/ml.

### Flow cytometry analysis

Lymphocytes were immunolabeled using a combination of CD4-Cy-chrome, (PE-Cy5, IgG1, clone RPA-T4) and CD8-Cy-chrome (PE-Cy5, IgG1, clone RPA-T8) antibodies to discriminate among the T cell populations. CD45RA- fluorescein isothiocyanate (FITC, clone HI100, IgG2b) and CD45RO-phycoerythrin (PE, clone UCHL1, IgG2a) (BD-Biosciences, Franklin Lakes, NJ, USA) were used to define cellular differentiation status and dual-color staining with CD8 (FITC, IgG1, clone MCD8) and CD38 (PE, IgG1, clone T16) or CD3 (FITC, IgG2b, clone UCHT1) and HLA-DR (PE, IgG2a, clone BRA30) (IQ Products, Groningen, The Netherlands) antibodies to analyze cellular activation. Five microliters of each antibody were distributed among the sample tubes, and 50 µl of fresh peripheral blood (in EDTA) was added. The samples were then mixed gently and incubated for 30 min at room temperature in the dark. Cell lysis was performed by adding 450 µL of FACS Lysing solution™ (BD Biosciences). Samples were then washed twice in PBS solution and fixed with PBS plus 1% paraformaldehyde, prior to acquisition on a FACSCalibur (BD Biosciences). At least 10.000 events were acquired in a lymphocyte gate, and phenotypic analysis was carried out using CellQuest™ software (BD). The percentages of CD38+ and HLA-DR+ cells were determined for the CD8+ and CD3+ T cell populations, respectively.

For CD3+/CD4+ and CD3+/CD8+ T cell subpopulations, the single-platform method was used to determine absolute counts. Briefly, 20 µl of TriTEST (BD-Biosciences) three-color (CD4-FITC, CD8-PE, and CD3-PerCP) antibodies and 50 µl of whole blood were added to bead-containing TruCount tubes (BD Biosciences). These were incubated for 15 min at room temperature before 450 µl of FACS Lysing solution™ was added. Samples were analyzed within 1 hour.

## Results

### Epidemiological, clinical and T cell immunological parameters of the groups studied

Forty-two (30 men and 12 women) HIV/leprosy-coinfected individuals with a median age of 39.5 (range: 21–69 years) were evaluated at the time of leprosy diagnosis. The diagnosis of leprosy was determined subsequent to the detection of HIV positivity in 40 of these individuals. Nearly half of these patients were diagnosed with leprosy prior to undergoing HAART while the other half was diagnosed as leprosy patients after HAART. In light of the classification criteria proposed by Talhari et al [25] for HIV/leprosy coinfected patients, the first half would be defined as having *M.* leprae-HIV true coinfection while the second half would be considered as having HAART-related leprosy.

According to the clinical status of leprosy and the absence of any RR symptoms by way of the Ridley and Jopling classification system, 26 of the 42 HIV/leprosy patients (61.9%) presented the borderline tuberculoid (BT) form, while 3 individuals (7.1%) presented the undetermined form, corresponding to the paucibacillary patients. With respect to the multibacillary patients, 6 (14.3%) were mid-borderline (BB), 4 (9.4%) presented the borderline lepromatous form (BL), and 3 (7.1%), the lepromatous (LL) form. For the multibacillary patients, the initial bacillary index median was 1.75, ranging from 0.5 to 4.83. Neurological impairment was absent in 64.3% of the patients at the outset of treatment. Among the 7 leprosy non-HIV patients, different clinical profiles were observed, including BT, BB, and LL.

Indeed, 35 of the 42 HIV/leprosy individuals were under HAART at the time of enrollment, the majority (57.1%) being administered the 3-drug regimen including zidovudine, lamivudine, and efavirenz, following the Brazilian Ministry of Health guidelines. The other patients were under other therapeutic schemes that included protease inhibitors. However, no differences were observed in the VL levels of patients administered different HAART regimens.

In order to evaluate T cell immunological parameters, all 85 individuals were primarily evaluated to quantify their CD3+/CD4+ and CD3+/CD8+ T cell subpopulations. The median absolute counts are presented in Table 1. The HIV/leprosy-infected individuals showed a significant reduction in CD4+ T cell absolute counts (*p*<0.05) regardless of VL levels (group 1 median: 336 cells/mm^3^; group 2 median: 341 cells/mm^3^; group 3 median: 285 cells/mm^3^) in comparison to the leprosy patients (median: 1079 cells/mm^3^) and the healthy controls (median: 813 cells/mm^3^). This CD4+ T cell absolute reduction is a typical characteristic of HIV infection. No differences in the CD4+ T cell absolute count distribution profiles were detected among the HIV/leprosy-coinfected and HIV-monoinfected groups.

**Table 1:** Median and interquartile range (IQR) of T cell immunophenotyping percentage values or absolute counts obtained from HIV/leprosy individuals according to viral load (VL) values and from HIV-monoinfected, leprosy patients and healthy controls.

	HIV/Leprosy			HIV-monoinfected		Leprosy	Healthy Controls
	Group 1	Group 2	Group 3	VL<LD	VL>LD	(n = 7)	(n = 20)
	(n = 24)	(n = 12)	(n = 6)	(n = 10)	(n = 6)		
CD3+/CD4+	336 (265–525)	341 (260–488)	285 (141–428)*	392 (379–450)	188 (109–300)	1079 (500–1533)* ^,^ † ^,^ ‡	813 (703–942)* ^,^ † ^,^ ‡
CD3+/CD8+	785 (462–1032)	734 (481–944)	921 (730–1303)	907 (792–1288)	1410 (1214–1864)* ^,^ †	542 (303–608)* ^,^ ‡	488 (410–573)* ^,^ † ^,^ ‡
CD4+/CD45RA+	22.4 (16.3–32.9)	37.7 (24.0–42.2)*	22.8 (15.4–33.3)	25.8 (19.0–31.6)	27.7 (20.2–33.0)	21.6 (16.7–27.5)	31.8 (19.8–44.9)
CD4+/CD45RO+	66.1 (55.1–80.5)	63.1 (53.4–71.6)	53.9 (46.7–63.3)	64.6 (59.6–75.1)	76.6 (70.9–83.2)	71.7 (58.8–75.6)	58.8 (45.6–74.6)
CD8+/CD45RA+	37.6 (32.5–40.8)	46.2 (40.0–50.5)	27.3 (22.6–41.3)	44.4 (40.1–48.3)*	36.8 (26.7–53.6)†	45.7 (3773–49.4)	47.4 (42.5–62.0)*
CD8+/CD45RO+	51.5 (41.0–64.2)	53.8 (45.5–65.0)	54.6 (44.9–64.7)	54.4 (42.9–62.4)	68.5 (40.5–81.3)	39.4 (31.5–48.1)	46.7 (34.0–54.2)
CD3+/HLA-DR+	19.3 (13.4–25.2)	16.4 (10.6–19.1)	39.3 (25.5–58.5)* ^,^ †	13.2(9.9–17.4)‡	13.8 (9.2–18.5)‡	5.8 (4.9–19.8)‡	8.4 (6.5–13.9)* ^,^ † ^,^ ‡
CD8+/CD38+	28.9 (15.1–37.5)	37.2 (26.0–68.3)	66.7 (60.2–80.4)*	53.3 (47.2–55.8)*	59.3 (57.7–64.6)*	26.1 (23.0–33.4)‡	8.7(4.2–13.4)* ^,^ † ^,^ ‡

*p<0,05 – Mann-Whitney *U* test: groups *versus* Group 1 HIV/leprosy individuals (VL<LD);

† *p*<0,05 – Mann-Whitney *U* test: groups *versus* Group 2 HIV/leprosy individuals (VL≥80<10,000 copies/ml);

‡ *p*<0,05 – Mann-Whitney *U* test: groups *versus* Group 3 HIV/leprosy individuals (VL≥10,000 copies/ml);

LD: limit of detection.

For CD8+ T cell absolute counts, lower values (*p*<0.01) were observed in group 1 (median: 783 cells/mm^3^) and group 2 (median: 734 cells/mm^3^) of the HIV/leprosy patients relative to the VL≥LD HIV-monoinfected ones (median: 1410 cells/mm^3^). Furthermore, HIV/leprosy group 1 had higher CD8+ T cell counts (*p*<0.05) when compared to those of the leprosy patients (median: 542 cells/mm^3^). As expected, all HIV/leprosy patients had significantly higher CD8+ T cell absolute counts (*p*<0.05) than the healthy individuals (median: 488 cells/mm^3^).

Cellular differentiation status was investigated by analyzing the surface expression of the CD45RA and CD45RO molecules in both CD4+ and CD8+ subpopulations (Table 1). For CD4+ T cells, frequency differences among CD45RA+ circulating cells were observed only when comparing groups 1 and 2 of the HIV/leprosy patients (medians: 22.4%, versus 37.7%; *p*<0.05), with the latter group showing a higher percentage of circulating cells. On the other hand, the CD45RO+ fraction of CD4+ T cells did not show any statistical differences among the study groups (Table 1).

On the other hand, the CD45RA+ fraction of the CD8+ T cells was found to differ among the groups (Table 1). HIV/leprosy group 1 had a lower percentage of CD45RA+ cells than the VL<LD HIV-monoinfected and healthy individuals (medians: 37.6%, versus 44.4% and 47.4%, respectively; *p*<0.05). On the other hand, the group 2 HIV/leprosy individuals had significantly more CD8+CD45RA+ T cells than the VL≥LD HIV-monoinfected individuals (medians: 46.2%, versus 36.8%; *p*<0.0001). No differences were observed in the proportion of circulating CD45RO+ CD8+ T cells among the various study groups, however.

Activation parameters were also evaluated in this study, including the total CD3+ T cell numbers, HLA-DR expression, and, for CD8+ cells, the expression of CD38. Because the expression of these activation markers, especially CD38, correlates strongly with HIV replication and viral load levels [19], [20], the HIV/leprosy-coinfected individuals were also evaluated according to their different VL levels.

**Figure 1:**
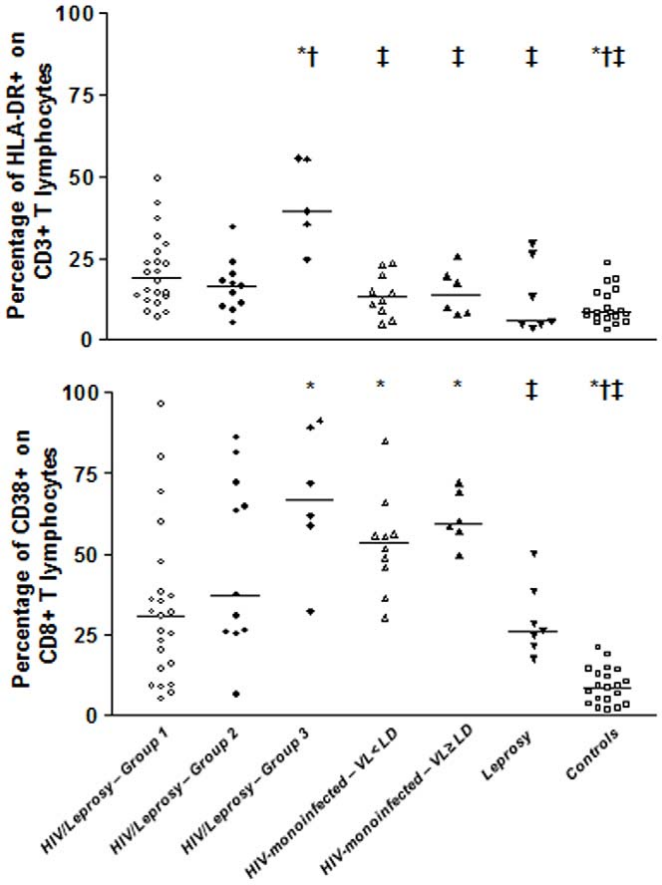
The percentage of HLA-DR+ in CD3+ T lymphocytes (*upper panel*) and the percentage of CD38+ in CD8+ T lymphocytes (*lower panel*) in samples from HIV/leprosy patients from group 1 (VL<LD), group 2 (VL≥80<10,000 copies/ml), and group 3 (VL≥10,000 copies/ml); HIV-monoinfected VL<80 copies/ml and VL>80 copies/ml patients; leprosy-infected individuals; and healthy controls. Bars represent median values. * *p*<0.05 – Mann-Whitney *U* test for each group compared with HIV/leprosy patients from group 1; † *p*<0.05 – Mann-Whitney *U* test for each group compared with HIV/leprosy patients from group 2; ‡ *p*<0.05 – Mann-Whitney *U* test for each group compared with HIV/leprosy patients from group 3. VL: HIV viral load. LD: Limit of detection.

As shown in Table 1 and Figure 1, a high frequency of CD3+ T cells expressing HLA-DR was observed in group 3 HIV/leprosy-infected individuals, in contrast to group 1 and group 2 (medians: 39.3%, versus 19.3% and 16.4%, respectively; *p*<0.05). In addition, higher levels of HLA-DR expression were also observed in groups 1 and 2 HIV/leprosy patients compared to the healthy individuals (median: 5.8%; *p*<0,05).

Similarly, differences were also observed in the circulation of CD8+ T cells expressing CD38 (Table 1, Figure 1). A high percentage of these cells was observed in group 2 HIV/leprosy patients (median: 66.7%) in contrast to the leprosy group and healthy controls (medians: 26.1% and 8.7%, respectively; *p*<0.01). However, this expression was not significantly higher than that seen in the VL<LD and VL≥LD HIV-monoinfected groups (median: 53.3% and 59.3%, respectively). When the HIV/leprosy patients had their HIV viral load controlled, their activation status was reduced (group 1 median: 30.7%; *p*<0.05), and this reduction was significant relative to that of the VL<LD and VL≥LD HIV-monoinfected patients. The activation levels in HIV/leprosy group 1 were significantly higher than those among the healthy controls (*p*<0.0001), but not the leprosy patients.

### Analysis of the IRIS/RR phenomenon

During the study, 9 of the 42 (21.4%) HIV/leprosy patients were identified as IRIS/RR by way of clinical status. Prior to the development of IRIS/RR symptoms, 7 were classified as BT while only 2 (patients Hs040 and Hs 045) were defined as BB.

All patients were under HAART during reaction although the time that lapsed between HAART submission and the onset of IRIS/RR varied from 3 months of treatment to 3 years post-treatment. Of interest, we were also able to quantify the CD4+ T cells and VL levels of 6 of the 9 IRIS/RR patients, previously to HAART submission. The CD4+ T cell absolute counts were under 350 cells/mm^3^, with a median value of 114 cells/mm^3^ (range: 17–318 cells/mm^3^). At the same time, high levels of VL were obtained, ranging from 20,000 to 5,700,000 copies/ml (median: 148,600 copies/ml). These patients were highly immunocompromised at baseline accompanied by intense viral replication. However, at the time of IRIS/RR, VL was virtually undetectable in 7 of the HIV/leprosy patients, except for 2 who had 320 (Hs012) and 4,880 copies/ml (Hs043). Indeed, as expected, their immunological status was restored since all 9 patients presented incremented CD4+ T absolute counts (medina: 408 cells/mm^3^ (range: 140–767 cells/mm^3^).

The other T cell immunological parameters were also evaluated in these 9 patients and although no clear pattern of expression was identified in terms of either cellular differentiation or activation markers in comparison with the HIV/leprosy patients with no RR signals, the IRIS/RR patients had high cellular activation levels at the onset of IRIS/RR. We were able to monitor these patients at different time points, including the post-IRIS/RR resolution moment, and interesting results were observed.

**Figure 2:**
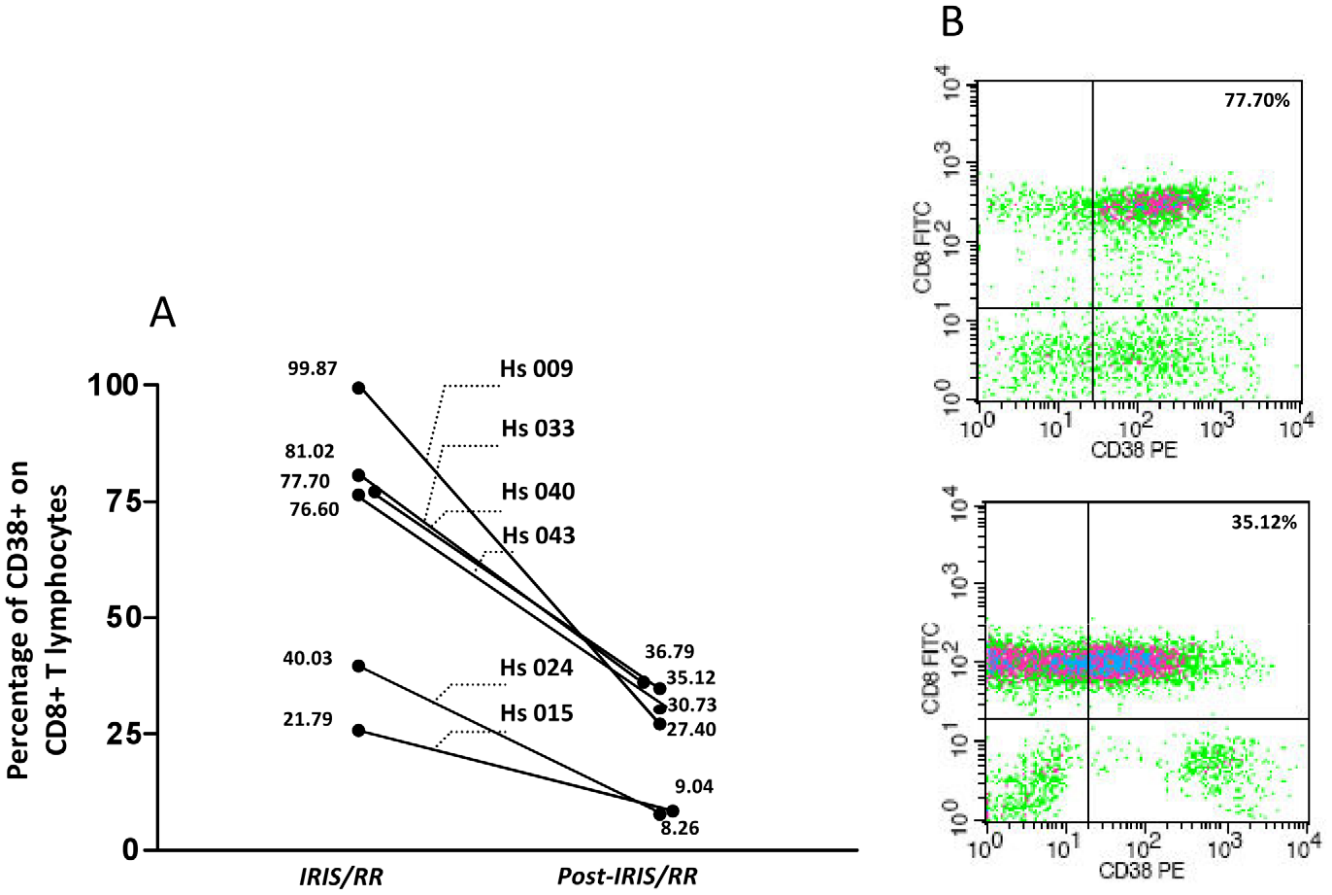
The percentage of CD38+ in CD8+ T lymphocytes obtained from HIV/leprosy-coinfected patients during the development of immune reconstitution inflammatory syndrome/reversal reaction (IRIS/RR) episodes and at the completion of reaction treatment with prednisone (Post-IRIS/RR) (Panel A). * *p*<0.05 – Wilcoxon *U* test. Representative flow cytometry profile presented by one HIV/leprosy coinfected patient (Hs040), during an IRIS/RR episode (upper dot plot) and post-IRIS/RR (under dot plot) evaluated for the expression of CD38 in CD8+ T lymphocytes (Panel B).

Six patients were analyzed at the initiation of an IRIS/RR episode and after the completion of treatment with prednisone. Regarding the expression of CD38 in CD8+ T cells, 6 individuals showed high levels of cellular activation (median: 77.15%; CI: 37.66%–96.13%) at the onset of IRIS/RR (Figure 2A). These values dropped significantly (*p*<0.05) post-IRIS/RR after 6 months of therapy with prednisone (median: 29.70%; CI: 11.17%–37.94%). Subsequent to prednisone treatment, cellular activation levels were significantly lower than those seen in the VL<LD HIV-monoinfected patients (*p*<0.01), similar to those detected in the leprosy patients but higher than that observed among the healthy controls (*p*<0.05). This reduction in CD38 expression in CD8+ T cells was clearly seen during flow cytometry analysis as observed by the cellular migration from the upper left panel to the upper right panel of the dot-plot graphics in Figure 2B.

After prednisone treatment, CD8+/CD38+ T circulating cell levels were higher in those HIV/leprosy patients who also had the highest activation levels during IRIS/RR, specifically individuals Hs009, Hs033, Hs040, and Hs043 (Fig. 2A), in comparison to the Hs015 and Hs024 patients, whose activation levels were lower at both time points.

Moreover, it is noteworthy that patient Hs009, who was also evaluated almost 4 months prior to the initiation of an IRIS/RR episode, already expressed CD38 in 59.82% of the circulating CD8+ T cells. Similarly, patient Hs019, analyzed just 5 days before the development of IRIS/RR symptoms, expressed CD38 in 77.02% of the CD8+ cells in contrast to the onset of IRIS/RR, when the percentage was 86.18%. Therefore, it can be assumed that an increase in these activated cells occurred prior to the IRIS/RR episodes.

Patient Hs012 was evaluated twice within a one-month period post-IRIS/RR. At the completion of prednisone therapy (post-IRIS/RR), 25.70% of the T cells were activated CD8+/CD38+ cells. However, one month later, this number had risen to 62.54%. This patient is still under surveillance; but, to date, no further IRIS/RR episodes have been detected.

## Discussion

Leprosy remains a significant global health problem, and over 40,000 new cases were registered in Brazil in 2009 [26]. Leprosy and HIV are two chronic infectious diseases with an overlapping geographic distribution in Brazil. Nonetheless, valuable information regarding the impact of these two diseases on T cell phenotypes in infected individuals remains scarce.

Immunological interactions between leprosy and HIV have been a topic of great interest over the last two decades or so. While an increase in the immunosuppressive form of leprosy (LL) was expected to occur in HIV/leprosy coinfected patients, this was not confirmed by follow-up studies. In contrast, occurrences of the tuberculoid form of leprosy predominate among these coinfected individuals whatever the T lymphocyte count in the blood [27], [28]. This was also observed at the present study, where 61.9% of the 42 HIV/leprosy coinfected patients presented the BT form of leprosy, while only 13 of them were identified as multibacillary cases. In the same way and also in contrast to the initially expected for leprosy and HIV coinfection [29], we observed low frequency of neurological impairment among the HIV/leprosy patients.

The most interesting phenomenon associated with the interaction between HIV and leprosy infection is the higher incidence of reversal reactions (RR) [28], suggesting that the immune regulation of each disease is independent. The higher frequency of RR after initiating HAART has brought on new challenges to the full understanding of this immunological phenomenon, previously been thought to be an *M. leprae*-specific immune reactivation. In addition, many of the leprosy cases appearing after the introduction of HAART with unusually inflammatory features have been classified as IRIS. We were able to identify 9 cases of IRIS/RR phenomenon among our HIV/leprosy coinfected patients. These individuals presented the symptoms of leprosy reversal reaction, after the onset of antiretroviral therapy and were considered among the patients having HAART-related leprosy [25]. Considering the same IRIS criteria previously used by our group to define IRIS cases [13], 93 out of 9 IRIS/RR patients (Hs012, Hs024 and Hs043) can be really defined as IRIS cases, since they developed the symptoms during the first 6 months of HAART . However, others studies also identified IRIS cases at periods of longer than 10 months [30]. In this study, we aimed to characterize some of the key T cell phenotypes involved in the HIV/leprosy interaction in coinfected vs. non-coinfected patients and analyze some major features of the IRIS/RR phenomenon.

We first examined CD4+ T cell counts, the classic immunological parameter for HIV infection follow-up. All HIV/leprosy-coinfected individuals showed a reduction in CD4+ cells, as expected for HIV- positive individuals, although the median values were similar or higher than those shown in other studies, regardless of viral load levels and/or HAART status [22], [23], [31]. An increase in absolute CD4+ T counts was observed in HIV/leprosy coinfected patients with undetectable VL levels, reflecting the quantitative immune reconstitution that occurs as a consequence of viral replication control and antiretroviral therapy. Moreover, despite the presence of *M. leprae*, HIV/leprosy coinfection exhibited the same CD4+ T cell behavior observed in HIV-infected patients in the absence of detectable VL under HAART, consistent with previous studies [23], [24], [31].

The lower levels of CD3+CD8+ T cells observed in groups 1 and 2 HIV/leprosy patients could be also attributed to a more effective control of viremia in these as opposed to the VL>LD HIV-monoinfected individuals. These data do not agree with the more severe immunopathology for HIV/leprosy coinfected individuals, previously suggested by Carvalho et al [31], probably due our analysis in terms of different viral load levels. On the other hand, even with a controlled viral load, when the group 1 was compared with the leprosy patients and healthy controls a higher percentage of CD8+ T cells was found. This data would suggest the effect of residual viremia on the immune system, undetectable by the currently methodology.

It is important to note that peripheral blood CD4+ or CD8+ T cell counts do not necessarily reflect either the number or function of these T cells at the actual sites of coinfection, although the evaluation of skin lymphocytic infiltrate in HIV/leprosy coinfected patients demonstrated the predominant involvement of CD8+ T cells at the site of disease, in granuloma formation, being more frequent than CD4+ T cells [32].

The CD4+ T cells that express the cell-surface isoforms CD45RA and CD45RO are major targets in HIV infection [33]. We evaluated the expression of these molecules by CD4+ and CD8+ circulating T cells, in order to define a cellular differentiation profile, but low differences were observed among the studied groups and we were not able to identify any defined profile. So, this was an important limitation of the present study, since in order to well define the differentiation pathway for T cells, many others surface molecules should be investigated [34], [35]. So, these differentiation profiles are currently being investigated in more detail by our team to better understand naïve cell behavior, central and effector memory cells, and other intermediary T cell fractions in HIV/leprosy coinfection scenario.

The evaluation of the activation parameters showed high frequencies of HLA-DR expression in CD3+ T cells and CD38+ expression in CD8+ T cells in HIV/leprosy-coinfected individuals, as was previously observed by the present authors in Leishmania/HIV-coinfected patients [24]. The HLA-DR molecule is capable of presenting antigenic peptides to the CD4+ T cell receptor complex and CD38 may function as an adhesion molecule. During HIV infection, changes in the expression of these markers are associated with immune dysfunction and disease progression. In our study, these expression frequencies, especially CD38 on CD8+ T cells, appeared to be associated with viral load because group 3 HIV/leprosy individuals showed the highest activation profile. However, frequent expression of these markers in the undetectable viral load group was also detected. Previous reports have shown that low levels of viral production have been known to persist in patients regardless of their being under long-term, effective HAART and that this remaining viremia could have an important impact on the immune system of some patients undergoing continuous, effective HAART [36]. Other studies have also suggested that even those patients with HAART-mediated viral load suppression show a high percentage of activated T cells and that this immune activation might be determined by immunological memory cells [37]. Our observation that T cell activation markers increased in HIV/leprosy cases despite low or undetectable HIV RNA levels might also be explained by the fact that immune activation could be determined by *M. leprae* in addition to HIV RNA levels. How the two pathogens act synergistically to drive immune activation remains to be fully elucidated.

The pathogenesis of IRIS in leprosy has not yet been clearly defined, but the re-establishment of CD4+ and CD8+ T lymphocytes after HAART may explain their development. Previous studies have shown that cellular responses to some mycobacterial diseases, such as *M. avium* complex infection, can be restored after 2 weeks of HAART in HIV1–infected patients [38]. HIV/tuberculosis patients also show inflammatory episodes characterized as IRIS after the initiation of HAART [39]. The increase in CD8+ T cell counts after HAART, independent of the CD4+ T cell count, may be an explanation for some of these reactions. Previous studies have demonstrated that the CD8+ T cell count was a risk factor associated with the development of herpes zoster after initiation of HAART [40]. Other authors have proposed that the increase in CD8+ T cells after HAART may result in clinical hepatitis caused by HBV as well as HCV [41], [42].

Increases in the expression of activation markers in effector CD8+ T cell populations may also provide insight into the immunopathogenesis of IRIS. Effector CD8+ T cells exhibit specialized functions such as cytotoxicity, antiviral cytokine production, telomerase activity, and the production of cytokines such as IL-2, IFN-γ, TNF, perforin, and granzymes. Of these, IFN-γ is known to increase in response to mycobacterial antigens in patients who developed tuberculosis/IRIS [37]. Once these cells are distributed in the periphery, they take on an immediate effector function and, thus, may be able to promote a rapid, but unsustained, response to peripheral antigens.

Interestingly, the CD38 antigen, a cellular activation marker previously associated with HIV pathogenesis [43], was significantly elevated in the CD8+ T cells of IRIS/RR individuals in our study but diminished after prednisone therapy. Of the 9 HIV/leprosy patients identified as experiencing IRIS/RR, 6 had been found to fit this profile, and 2 were obviously undergoing IRIS at the time of reaction. The percentage of CD8+/CD38+ T cells increased prior to the initial onset of IRIS/RR symptoms. These data open a promising channel of investigation into CD38 expression, which could be a useful biomarker for identifying coinfected individuals with activated immune systems at risk of developing IRIS/RR. Obviously, the evaluation of the leprosy individuals under reversal reaction need to be conducted, in order to investigate if the CD38 expression on CD8+ T cells will follow the same profile.

In summary, leprosy and HIV lead to some similar and some distinct immune alterations. In HIV-monoinfected and HIV/leprosy coinfected individuals with high viremia, CD4+ T cell depletion together with pronounced T cell activation may be involved in a generalized hyporesponsiveness of the immune system. In coinfected subjects, HIV apparently controls the overall immunological scenario, especially in patients with low CD4+ T counts. HIV and leprosy seem to have no additive effects in that each infection causes specific immune changes, which do not appear to contribute to the synergistically unfavorable effects of coinfection.

Our findings regarding IRIS/RR individuals may prove useful in designing future studies aimed at identifying patients at risk of developing IRIS/RR. Future prospective pathogen-specific IRIS immunological studies will likely be required to increase our understanding of its pathogenesis and to identify potential biomarkers for the disease.

## References

[1] Hogg RS, O'Shaughnessy MV, Gataric N, Yip B, Craib K (1997). Decline in deaths from AIDS due to new antirretrovirals.. Lancet.

[2] Palella FJ, Delaney KM, Moorman AC, Loveless MO, Fuhrer J (1998). Declining morbidity and mortality among patients with advanced human immunodeficiency virus infection. HIV Outpatient Study Investigators.. N Engl J Med.

[3] Jehle AW, Khanna N, Sigle JP, Glatz-Krieger K, Battegay M (2004). Acute renal failure on immune reconstitution in an HIV-positive patient with miliary tuberculosis.. Clin Infect Dis.

[4] Race EM, Adelson-Mitty J, Kriegel GR, Barlam TF, Reimann KA (1998). Focal mycobacterial lymphadenitis following initiation of protease-inhibitor therapy in patients with advanced HIV-1 disease.. Lancet.

[5] Koval CE, Gigliotti F, Nevins D, Demeter LM (2002). Immune reconstitution syndrome after successful treatment of Pneumocystis carinii pneumonia in a man with human immunodeficiency virus type 1 infection.. Clin Infect Dis.

[6] Shelburne SA, Hamill RJ, Rodriguez-Barradas MC, Greenberg SB, Atmar RL (2002). Immune reconstitution inflammatory syndrome: emergence of a unique syndrome during highly active antiretroviral therapy.. Medicine (Baltimore).

[7] Murdoch DM, Venter WD, Van Rie A, Feldman C (2007). Immune reconstitution inflammatory syndrome (IRIS): review of common infectious manifestations and treatment options.. AIDS Res Ther.

[8] Cheng VC, Ho PL, Lee RA, Chan KS, Chan KK (2002). Clinical spectrum of paradoxical deterioration during antituberculosis therapy in non-HIV-infected patients.. Eur J Clin Microbiol Infect Dis.

[9] Lawn SD, Wood C, Lockwood DN (2003). Borderline tuberculoid leprosy: an immune reconstitution phenomenon in a human immunodeficiency virus-infected person.. Clin Infect Dis.

[10] Couppie P, Abel S, Voinchet H, Roussel M, Helenon R (2004). Immune reconstitution inflammatory syndrome associated with HIV and leprosy.. Arch Dermatol.

[11] Visco-Comandini U, Longo B, Cuzzi T, Paglia MG, Antonucci G (2004). Tuberculoid leprosy in a patient with AIDS: A manifestation of immune restoration syndrome.. Scand J Infect Dis.

[12] Pignataro P, Rocha Ada S, Nery JA, Miranda A, Sales AM (2004). Leprosy and AIDS: Two cases of increasing inflammatory reactions at the start of highly active antiretroviral therapy.. Eur J Clin Microbiol Infect Dis.

[13] Menezes VM, Sales AM, Illarramendi X, Miranda A, Gonçalves Morgado M (2009). Leprosy reaction as a manifestation of immune reconstitution inflammatory syndrome: a case series of a Brazilian cohort.. AIDS.

[14] Kharkar V, Bhor UH, Mahajan S, Khopkar U (2007). Type I lepra reaction presenting as immune reconstitution inflammatory syndrome.. Indian J Dermatol Venereol Leprol.

[15] Sasaki S, Takeshita F, Okuda K, Ishii N (2001). Mycobacterium leprae and leprosy: a compendium.. Microbiol Immunol.

[16] Scollard DM, Smith T, Bhoopat L, Theetranont C, Rangdaeng S (1994). Epidemiologic characteristics of leprosy reactions.. Int J Lepr Other Mycobact Dis.

[17] French MA, Lenzo N, John M, Mallal SA, McKinnon EJ (2000). Immune restoration disease after the treatment of immunodeficient HIV-infected patients with highly active antiretroviral therapy.. HIV Med.

[18] Jevtovic DJ, Salemovic D, Ranin J, Pesic I, Zerjav S (2005). The prevalence and risk of immune restoration disease in HIV-infected patients treated with highly active antiretroviral therapy.. HIV Med.

[19] Autran B, Carcelain G, Li TS, Blanc C, Mathez D (1997). Positive effects of combined antiretroviral therapy on CD4+ T cell homeostasis and function in advanced HIV disease.. Science.

[20] Kaushik S, Vajpayee M, Sreenivas V, Seth P (2006). Correlation of T-lymphocyte subpopulations with immunological markers in HIV-1-infected Indian patients.. Clin Immunol.

[21] Benito JM, Lopez M, Lozano S, Martinez P, Gonzaez-Lahoz J (2004). CD38 expression on CD8(+) T lymphocytes as a marker of residual virus replication in chronically HIV-infected patients receiving antiretroviral therapy.. Aids Research and Human Retroviruses.

[22] Benito DM, Lopez M, Lozano S, Ballesteros C, Martinez P (2005). Differential upregulation of CD38 on different T-cell subsets may influence the ability to reconstitute CD4(+) T cells under successful highly active antiretroviral therapy.. Jaids-Journal of Acquired Immune Deficiency Syndromes.

[23] Appay V, Sauce D (2008). Immune activation and inflammation in HIV-1 infection: causes and consequences.. J Pathol.

[24] Santos-Oliveira JR, Giacoia-Gripp CBW, Oliveira PA, Sabagga-Amato V, Lindoso JA (2010). High levels of T lymphocytes activation are observed in Leishmania-HIV-1 co-infected despite low HIV viral load.. BMC Infect Dis.

[25] Talhari C, Mira MT, Massone C, Braga A, Chrusciak-Talhari A (2010). Leprosy and HIV coinfection: a clinical, pathological, immunological, and therapeutic study of a cohort from a Brazilian referral center for infectious diseases.. J Infect Dis.

[26] Portal da Saúde website.. http://portal.saude.gov.br/portal/arquivos/pdf/graf_1_n_percentual_casos_hanse_2009_01_12.pdf.

[27] Sampaio EP, Caneshi JR, Nery JA, Duppre NC, Pereira GM (1995). Cellular immune response to *Mycobacterium leprae* infection in human immunodeficiency virus-infected individuals.. Infect Immun.

[28] Sarno EN, Illarramendi X, Nery JAC, Sales AM, Gutierrez-Galhardo MC (2008). HIV-M. leprae interaction: can HAART modify the course of leprosy?. Public Health Rep.

[29] Lockwood DN, Lambert SM (2011). Human immunodeficiency virus and leprosy: an update.. Dermatol Clin.

[30] Massone C, Talhari C, Ribeiro-Rodrigues R, Sindeaux RH, Mira MT (2011). Leprosy and HIV coinfection: a critical approach.. Expert Rev Anti Infect Ther.

[31] Carvalho KI, Maeda S, Marti L, Yamashita J, Haslett PA (2008). Immune cellular parameters of leprosy and human immunodeficiency virus-1 co-infected subjects.. Immunology.

[32] Massone C, Talhari C, Talhari S, Brunasso AM, Campbell TM (2008). Immunophenotype of skin lymphocytic infiltrate in patients co-infected with Mycobacterium leprae and human immunodeficiency virus: a scenario dependent on CD8+ and/or CD20+ cells.. Br J Dermatol.

[33] Woods TC, Roberts BD, Butera ST, Folks TM (1997). Loss of inducible virus in CD45RA naive cells after human immunodeficiency virus-1 entry accounts for preferential viral replication in CD45RO memory cells.. Blood.

[34] Decrion AZ, Varin A, Drobacheff C, Estavoyer JM, Herbein G (2007). A subset of functional effector-memory CD8+ t lymphocytes in human immunodeficiency virus-infected patients.. Immunology.

[35] Sallusto F, Geginat J, Lanzavecchia A (2004). Central memory and effector memory T cell subsets: function, generation, and maintenance.. Annu Rev Immunol.

[36] Havlir DV, Strain MC, Clerici M, Ignacio C, Trabattoni D (2003). Productive infection maintains a dynamic steady state of residual viremia in human immunodeficiency virus type 1-infected persons treated with suppressive antiretroviral therapy for five years.. J Virol.

[37] Marziali M, De Santis W, Carello R, Leti W, Esposito A (2006). T-cell homeostasis alteration in HIV-1 infected subjects with low CD4 T-cell count despite undetectable virus load during HAART.. AIDS.

[38] Bourgarit A, Carcelain G, Martinez V, Lascoux C, Delcey V (2006). Explosion of tuberculin-specific Th1-responses induces immune restoration syndrome in tuberculosis and HIV co-infected patients.. AIDS.

[39] Schluger NW, Perez D, Liu YM (2002). Reconstitution of immune responses to tuberculosis in patients with HIV infection who receive antiretroviral therapy.. Chest.

[40] Martinez E, Gatell J, Moran Y (2008). High incidence of herpes zoster in patients with AIDS soon after therapy with protease inhibitors.. Clin Infect Dis.

[41] Carr A, Cooper DA (1997). Restoration of immunity to chronic hepatitis B infection in HIV-infected patient on protease inhibitor.. Lancet.

[42] Rutschmann OT, Negro F, Hirschel B, Hadengue A, Anwar D (1998). Impact of treatment with human immunodeficiency virus (HIV) protease inhibitors on hepatitis C viremia in patients coinfected with HIV.. J Infect Dis.

[43] Liu Z, Cumberland WG, Hultin LE, Prince HE, Detels R (1997). Elevated CD38 antigen expression on CD8+ T cells is a stronger marker for the risk of chronic HIV disease progression to AIDS and death in the Multicenter AIDS Cohort Study than CD4+ cell count, soluble immune activation markers, or combinations of HLA-DR and CD38 expression.. J Acquir Immune Defic Syndr Hum Retrovirol.

